# Avian Gyrovirus 2 DNA in Fowl from Live Poultry Markets and in Healthy Humans, China

**DOI:** 10.3201/eid2108.150203

**Published:** 2015-08

**Authors:** Jianqiang Ye, Xiaoyan Tian, Quan Xie, Yu Zhang, Yuanzhao Sheng, Zhenwen Zhang, Chengming Wang, Hong Zhu, Yumeng Wang, Hongxia Shao, Aijian Qin

**Affiliations:** Ministry of Education Key Laboratory for Avian Preventive Medicine and Key Laboratory of Jiangsu Preventive Veterinary Medicine, Yangzhou University, Yangzhou, China (J. Ye, X. Tian, Q. Xie, Y. Zhang, Y. Sheng, H. Zhu, Y. Wang, H. Shao, A. Qin);; Jiangsu Co-innovation Center for Prevention and Control of Important Animal Infectious Diseases and Zoonoses, Yangzhou (J. Ye, Z. Zhang, C. Wang, H. Shao, A. Qin);; College of Medicine, Yangzhou University, Yangzhou (Z. Zhang)

**Keywords:** Avian gyrovirus 2, chicken anemia virus, viruses, live poultry market, zoonoses, China

**To the Editor:** In 2011, a chicken anemia virus (CAV)–related sequence, designated avian gyrovirus 2 (AGV2), was first identified in serum samples from diseased chickens in Brazil (*1*). During the same year, a human gyrovirus (HGyV) sequence that had high identity to AGV2 was detected in the skin of humans in France (*2*). As with CAV, 3 open reading frames (ORFs) for encoding viral proteins (VP) 1–3 (*2*) overlapped in genome of AGV2. Recently, HGyV/AGV2 has been detected in Hong Kong in chicken meat for consumption by humans, in human blood samples from donors in France, and in HIV-positive persons and organ transplant recipients in Italy and the United States (*3*–*5*). However, the epidemiology, host range, transmission route, and pathogenesis of AGV2 remain poorly understood. Bullenkamp et al. found that AGV2 VP3 protein, like CAV VP3, can induce apoptosis of tumor cells (*6*). Also, Abolnik et al. reported the detection in Southern Africa of AGV2 in brain tissue of chickens that showed severe neurologic signs (*7*). These findings highlight the potential pathogenesis of AGV2.

So far, little is known about AGV2 in mainland China among chickens and humans. Because live poultry market (LPMs) play a critical role in the transmission of poultry pathogens to humans, we used PCR to investigate the presence of AGV2 in chickens (54 feather shaft samples) from 4 LPMs in Yangzhou and in 178 human blood samples from healthy persons living in Yangzhou. The DNA from the feather shafts and human blood were extracted as previously described (*8*). PCR was performed by using the following 2 primers: AGV2_F 5′−CGTGTCCGCCAGCAGAAACGAC-′3 and AGV2_R 5′-GGTAGAAGCCAAAGCGTCCACGA-′3. The PCR targets partial VP2 and VP3 genes that have an expected size of 346 bp. The parameters of the PCR were as follows: 1 cycle at 95°C for 5 min; then 30 cycles at 94°C for 30 s, 64°C for 30 s, and 72°C for 30 s; and 1 cycle at 72°C for 10 min. PCR showed that a band with the size of ≈346 bp could be amplified in 10 of 54 chicken feather samples and in 2 of 178 human blood samples.

We confirmed the AGV2 specificity of these PCR–amplified bands by direct sequencing using the Sanger method. The sequence assay showed that the 12 sequences identified here had 98.3%–100% homology to each other and 92.2%–99.1% aa identity to AGV2 samples previously deposited in GenBank (see Figure legend for accession numbers). The positive rates for samples from the 4 LPMs tested were 25%, 12.5%, 15.8%, and 20%; the positive rate for the 178 human blood samples was 1.1%. The low positive frequency of AGV2 in human blood detected in this study is consistent with that found by investigation in other countries (*3*,*4*). Because the limit of detection of PCR in this study was estimated to be 2.7 copies of AGV2 DNA using dilutions of a plasmid with partial AGV2 sequence, we determined that the copy number of AGV2 in the 2 positive human blood samples was 2.7 × 10^3^ copies/mL plasma. 

We also constructed a phylogenetic tree using the neighbor-joining method (1,000 bootstrap replications) with MEGA6 (*9*). The tree analysis revealed that the 12 AGV2 isolates we identified and 7 AGV2 isolates from GenBank clustered into 2 subgroups on the basis of the PCR amplified fragment (Figure). The 12 AGV2 sequences we identified clustered together with gyrovirus sequences detected in ferret and human samples in subgroup I, and the prototype sequence Ave3 was located in subgroup II. The 12 AGV2 showed ≈92.2%–93% aa identity to Ave3, and <99.1% homology with isolates CL33, G13, and 915F06007 detected in ferret and human samples. The 12 AGV2 sequences also showed ≈93%–93.9% identities to ACV2 sequence that was previously identified in human fecal samples from mainland China (GenBank accession no. JQ690763). The China sequence also clustered with Ave3 in subgroup II. These findings indicate that ≥2 subgroups of AGV2 are circulating in mainland China.

**Figure:**
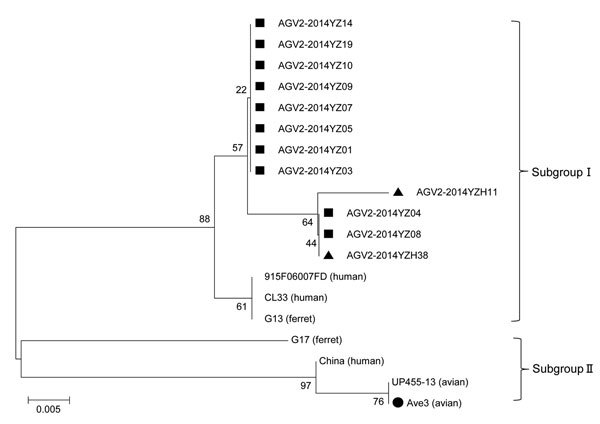
Phylogenetic analysis of AGV2. The phylogenetic tree was constructed by using the neighbor-joining method (1,000 bootstraps) with MEGA6 (*9*). Black squares indicate the 10 AGV2 identified from live poultry markets; black triangles indicate the 2 AGV2 identifed from human blood; black dot indicates the prototype AGV2 sequence. Sequences and GenBank accession nos.: AGV2–2014YZ01, KP993124; AGV2–2014YZ03, KP993125; AGV2–2014YZ04, KP993126; AGV2–2014YZ05, KP993127; AGV2–2014YZ07, KP993128; AGV2–2014YZ08, KP993129; AGV2–2014YZ09, KP993130; AGV2–2014YZ10, KP993131; AGV2–2014YZ14, KP993132; AGV2–2014YZ19, KP993133; AGV2–2014YZH11, KP993134; AGV2–2014YZH38, KP993135; 915 F 06 007 FD, FR823283; CL33, JQ308212; G13, KJ452214; G17, KJ452213; China, JQ690763; UP455–13, KF436510; Ave3, HM590588. Scale bar indicates amino acid substitutions per site.

Our results demonstrate the presence of AGV2 in LPMs and human blood in mainland China. The amplification and analysis of partial AGV2 sequences was the major limitation in our method. The high homology between sequences identified in LPMs and human blood indicates the LPMs are a potential source for AGV2 in humans. Unlike our 12 conserved AGV2, AGV2 identified by Santos et al. in southern Brazil varied <15.8%, and these variants of AGV2 were mainly detected in diseased chickens (*8*). However, little is known about the molecular epidemiology of these AGV2 variants in other countries. More recently, Varela et al. reported the detection of AGV2 in poultry vaccines, indicating the potential role of contaminated vaccines in the spread of AGV2 (*10*). Future studies should investigate the large geographic distribution of AGV2 and monitor the variants, the host range, and the associated diseases.
